# Supplementary material for: Are carbon emissions trading and green financial instruments synergistic? -Comprehensive quantitative research based on content analysis
Source: PLoS One. 2024 Mar 7;19(3):e0298601. doi: 10.1371/journal.pone.0298601 (PMC10919601; doi:10.1371/journal.pone.0298601)
Supplement: S1 Data — (ZIP) [file pone.0298601.s001.zip › Data/地级市Z_F工作报告.zip!/║┌┴·╜¡/║╫╕┌╩╨/└┤╫╘╣1⁄2╓┌║┼í╛┬φ┐╦╩2╛▌═°í┐_2011.docx]

各位代表：

　　现在，我代表市政府向大会作工作报告，请予审议。请政协委员和其他列席的同志提出意见。

　　一、“十一五”时期及2010年工作回顾

　　“十一五”时期是极不平凡的五年。特别是近三年，我们经历了百年不遇的国际金融危机，更经历了鹤岗有史以来最严重、最集中的旱灾、矿难、“集资借贷”事件，以及罕见的涝灾、雨灾和森林火灾。面对这一切，我们在省委、省政府和市委的正确领导下，牢牢把握改革、发展、稳定的大局，全力迎战自然灾害，有效处置突发事件，稳健驾驭复杂局面，不仅经受住了一系列严峻考验，而且圆满完成了“十一五”规划确定的各项任务目标，开创了经济社会发展的新局面。

　　过去的五年，是经济实力明显增强的五年。全市地区生产总值从111.2亿元增加到251亿元，是“十五”期末的2.3倍；人均地区生产总值从1254美元增加到3495美元，是“十五”期末的2.8倍；全口径财政收入从13.7亿元增加到37亿元，一般预算收入从5.7亿元增加到15.5亿元，均为“十五”期末的2.7倍。固定资产投资累计完成429亿元，是“十五”时期的3.6倍；社会消费品零售总额累计达到263亿元，是“十五”时期的1.9倍；城镇人均可支配收入从7489元增至12044元，是“十五”期末的1.6倍，2010年增幅列全省第三位；农民人均纯收入从3468元增至6300元，是“十五”期末的1.8倍，首次超过全国、全省平均水平。

　　过去的五年，是项目建设实效突出的五年。全市集中力量连续开展了两轮“项目建设年”活动，共实施千万元以上产业项目214个，完成投资近200亿元。特别是近三年，已开竣工的产业项目数，超过了改革开放以来近三十年的总和。2010年，我们组织开展了“项目攻坚年”和“四比”竞赛等活动，共实施投资千万元以上产业项目100个，其中投产40个，在建60个。煤电化项目完成布局。编制了煤电化基地总体规划和煤焦化副产品综合利用规划，两个大型甲醇项目已获批准；龙煤东化化肥项目完成总承包招标；华鹤大颗粒尿素项目已由中海油集团承接，并开始土建施工。四个百万吨煤焦化项目进展顺利，仅两年时间，鹤翔焦化投产，宝源焦化完成主体工程，征楠焦化开工建设，万昌焦化完成征地。鹤矿公司鸟山煤矿120万吨主焦煤项目、东北三省最大最先进的400万吨洗煤项目等开工建设，嘉晨180万吨洗煤、东兴120万吨洗煤、成金1.8亿块烧结砖、立达矸石热电90万千瓦一期项目投产。食品加工项目快速落实。新建7个共计170万吨稻米加工项目（其中30万吨4个）多数近期投产；万源200万吨水稻加工及综合利用项目分三期建设，获得省政府4500万元资金支持，并与上海恒诚投资公司签订合作上市协议，加工规模和深度居全国之首。兴汇集团百万吨玉米深加工二期项目（70万吨）正在安装设备，年产1.2万吨白酒项目投产；宝泉酱业5万吨豆瓣酱扩产改造和4个累计年产6.2万吨豆制品项目开工建设。北大荒集团与双汇集团合作的200万头生猪屠宰及年产1.5万吨熟食项目投产。农业部规划设计院玉米秸秆循环利用项目和“台湾农民创业园”项目开工建设。高新技术项目加快推进。拥有国家专利的台湾华升公司矿用救生舱项目、龙跃公司鲜青玉米剥皮机项目和香港南海油集团石墨深加工项目开工建设；与院校合作的禾友农药搬迁改造、三精生物制药扩产、双兰星制药项目投产。鹤矿鑫塔百万吨干法水泥项目完成主体工程。新能源项目顺利启动。望云峰5万千瓦风电项目投产；龙源解放山10万千瓦风电、泰丰和人和稻壳发电等项目开工建设，宝泉岭国能秸秆发电项目完成土地审批，等等。与此同时，经努力争取，我市省级“工业示范基地”获批，并被列入“黑龙江省级财源建设示范区”，县(区)园区也基本建成，结束了我市没有产业园区的历史。最近，我市又被国家列入《大小兴安岭林区生态保护与经济转型规划》，“鹤岗接替产业集聚区”等成为国家重点支持项目。近年来，全市上下抓项目建设的意识、热情、能力和成效都明显增强，态势越来越好。

　　过去的五年，是三次产业加快发展的五年。“十一五”期末，一二三产增加值实现60.2亿元、125.4亿元、65.4亿元，分别为“十五”期末1.6倍、2.3倍和1.5倍。工业整体素质明显增强，规模以上工业企业达到134户，比“十五”期末增加62户。2010年，围绕实现“双一”目标，开展了“两抓两保”攻坚战活动，举办了我市首届“星级企业”和名优产品评选活动，并对“国家级高新技术”和“中国驰名商标”企业进行了表彰。大力实施“精煤战略”，全市生产原煤1833万吨，生产洗煤1221万吨，实现税收18.5亿元，增长13.7%。其中，地方煤矿在连续三年完成省政府下达关闭27个矿井任务的情况下，生产原煤583万吨，实现税收5.33亿元，分别增长58.6%和40%。烟草、通讯、电力、啤酒、白酒等企业效益逐步提升，工业用电量增长7%。建筑业实现税收增长50%，商品房平均售价2160元/平方米，比“十五”期末增长62.6%。现代农业加快建设，粮食实际产量从9.2亿斤增至20.5亿斤，域内粮食总产从44.4亿斤增至71.2亿斤，分别是“十五”期末的2.2倍和1.6倍，创历史最高纪录。累计发放各类涉农补贴资金9亿元，是“十五”期间的4.2倍。实施各类农田水利工程项目122个，是“十五”时期的2.5倍。2010年，全市农作物播种面积达到325.5万亩，其中，水稻新增38万亩，当年实现了打造“百万亩水稻”的目标，占全省新增面积的1／6，受到省政府表彰。有机、绿色、无公害食品种植面积达155.6万亩，有机绿色标识达到220个。建成养殖基地12个，畜禽饲养量进一步增长。农业产业化加快推进，国家级、省级龙头企业达到12户，5万吨以上农产品加工企业达到83户；“梧桐”、“龙水”、“实实”牌大米被评为“中国驰名商标”，占全省的3／5；我市被评为全省“农业产业化先进市”。加强农业基础设施建设，完成了江萝灌区渠首、小鹤立河水库扩建等6个水利工程，总库容8.8亿立方米的关门嘴子大型水库被列入省重点工程。黑龙江流域现代农业科技示范园、马铃薯繁育基地和绥滨网箱养鱼基地等开工建设。争取农机直补资金2053万元，“农机汽车大市场”建成使用，农业生产综合机械化程度达到93%，场县共建跨区作业面积143万亩。新建水稻育秧大棚5115栋，大棚育秧率达到95%。农村改革扎实推进，完成了24个省级示范村建设任务，农民专业合作组织达到121个。第三产业迅速发展。抓住我市被列为“北国风光特色旅游开发区”“界江游”龙头的机遇，在两年多的时间里，自筹资金近3亿元，完成了黑龙江流域博物馆、“大荒情纪念馆”、金顶山石林公园、嘟噜河湿地公园、月牙湖风情园、界江国际大酒店等26个旅游开发项目，购置了7艘豪华游船。岭北露天矿区被命名为国家级矿山地质公园，黑龙江三峡森林公园、鹤岗森林公园、鹤北联营母树林公园被命名为国家级森林公园，名山岛、细鳞河、金顶山等5个景区被命名为国家3A或2A级景区。2010年，成功举办了“首届中国·黑龙江鹤岗国际界江旅游节”，以及“激情广场”、“殷秀梅家乡演唱会”等文化、体育、经贸活动。名山镇被列为全省旅游名镇和国家“边贸型重点扶持镇”，基础设施已开工建设。全面加强城市形象宣传，编制了《神奇鹤岗》、《魅力鹤岗风光》、《鹤岗邮票珍藏册》等宣传品；在央视《朝闻天下》、香港《大公报》、《中国旅游报》及公路、机场等地集中开展了宣传；“北国边疆文化旅游区”等项目被列入国家旅游“十二五”规划，我市被中国国际旅游协会等单位授予“中国最佳山水旅游胜地”称号。这一切，标志着我市旅游业进入了历史上最好的时期。不断强化促进消费政策措施，深入实施“商务惠农”工程，完成各类“家电下乡”销售额1.4亿元。社区“双进”工程取得新进展，有3个社区、2个企业分别被评为国家级、省级商业示范区（店）。积极引进大型企业，仅两年时间，上海红星·美凯龙、吉林温馨鸟、江海证券、哈尔滨银行、龙江银行等十几个企业落户营业。编制了《鹤岗市现代物流业发展规划》，8个物流园区项目开工建设。

　　过去的五年，是城乡面貌不断改善的五年。“十一五”时期，共建成高等级公路、城市道路和农村公路252公里、35公里和1340公里，分别比“十五”时期增长200%、47%和330%。完成绿化任务46万亩，森林覆盖率、建成区绿化覆盖率、绿地率、人均公园绿地面积分别达到44%、42%、40%和14.7平方米。水、电、气等城乡基础设施也逐步完善。2010年，我市全面掀起新一轮城乡建设高潮，明确了“宜居、宜业、宜游的滨水森林城市”定位，并全面加大城市规划、建设和管理力度，集中解决了一批群众反映强烈的突出问题，极大地改善了城市面貌。大力实施“畅通工程”，当年投资3亿多元，建成28条市内道路、6条主干线公路和5座桥梁。特别是六个城市出口路一次完成改造升级，运作多年的鹤名一级公路实质性开工；鹤佳、鹤名两条铁路已上报铁道部审批。大力实施“四供两治”工程，投资1.1亿元对城市水源地和供水管网进行了集中改造，城市日供水能力增加9万吨，水质明显提高；成立了水务抢险突击队，有效保证了城市供水防洪安全。供热三期工程竣工，新增供热面积360万平方米，完成了200多栋旧楼供热设施和18个小区集中供热改造工程，基本解决了“弃供”问题。西部污水处理厂竣工运行，东部污水处理、市医院医疗污水处理、生活垃圾无害化处理项目开工建设，结束了我市没有污水、垃圾处理工程的历史。进一步加快城乡电网建设步伐，实施了8个110千伏及以上输变电项目。大力实施“五化”工程，新建或改造了人民、振兴等14个城市广场和公园。加大硬化力度，完成裸土治理60多万平方米，铺设人行道板5万多平方米，新砌路边石53公里。加大绿化力度，首次实施春秋两季植树，完成植树238万株，基本完成了一次性绿化煤矿矿区、沉陷腾空区、裸露山体和村屯任务。加大净化力度，狠抓环境卫生，开展了“除冰雪、清垃圾和小区环境整治会战”等活动，一次性取缔5个白灰厂，关闭5个造纸厂，消灭了30个大烟囱和41个小锅炉；加大了对1400多栋“弃管楼”、“无管楼”治理力度。投入1000多万元，购置42台（套）环卫设备。实施了门前“四包”等制度，城市卫生环境明显改善。加大亮化力度，重点对城市道路、广场等进行了亮化升级改造，投入553万元，增加景观灯近300盏，全市亮灯率达到98%。加大美化力度，共拆除各类违规建筑房屋棚厦1000多个，全部更新了路街牌和交通指示标识等设施。争取1.6亿元资金（列全省第一位），完成了180万平方米既有建筑立面美容工程，并在全省既有建筑节能工作会上介绍了经验。大力实施“滨水城市”工程，完成了以“五湖一河”为重点的滨水城市规划，其中清源湖、天水湖、鹤立湖和小鹤立河沿岸景观带工程开工建设。此外，对城区13条河道特别是易灾区的防洪工程进行了大规模治理，基本解除了居民区的水淹威胁。大力实施城市功能提升工程，一年开工建设了黑龙江煤电化高层人才培训中心、国省级检测检验中心、公共资源公开交易中心、城市应急指挥中心、人民群众诉访调处中心、职业教育培训中心、煤矿安全技术培训中心、太平沟专家接待中心、市医疗卫生中心、动物疫病防控中心、文化活动中心、青少年科技活动中心，完善了全民健身中心。这13个中心建成后，对提升我市城市功能和品位具有重要意义。

　　过去的五年，是社会事业全面发展的五年。教育事业长足进步，全面实施素质教育，教学质量明显提高，建成省级标准化学校29所，高中阶段毛入学率达到86%，高考升学率达到86.5%；农村义务教育经费全部纳入财政保障范围；新建13所学校8.7万平方米教学设施，三中被确定为“沈阳军区选拔飞行员优质生源基地”。2010年，全面落实了中等职业学校农村贫困家庭学生和涉农专业学生等免费政策，启动了中小学校舍安全工程，翻建了东方红小学和兴安四校等校舍，现有城市学校D级危房改造任务全部完成。卫生服务日趋完善，医疗卫生体制改革全面展开，新建了疾病控制中心、传染病院、市医院急救中心及一批县乡社区医疗机构，社区卫生服务机构覆盖率达到90%。全市人口出生率控制在4.77‰、自然增长率控制在0.42‰。2010年，实施了“婴幼儿健康促进计划”和免费婚前医学检查等政策，建设了市人民医院和市妇幼保健院重症监护室、国家级流动人口监测站及16个基层卫生服务站（室），并在全国率先建立了特困计生家庭廉租房制度。新型农村合作医疗人均筹资标准达到150元，参合率达到100%。科技水平逐步提高，共实施高新技术项目408项，形成科技成果124项，获得国家级专利420项，列入省重大科技成果转化项目3项，科技成果转化率达到70.6%，比“十五”期间提高了10.5个百分点。2010年，我市有2户企业被确定为国家高新技术企业，有17户企业被确定为省级高新技术企业，“石墨工程技术研究中心”、“水稻深加工研究中心”、“农药中试基地”、“中药产品基地”等研发机构和68个农业科技园建成。文化建设成果显著，改造了市人民剧场、无线电视转播塔等一批基础设施，全市有线电视入户率达到52%。2010年，市博物馆完成主体工程，地质博物馆开馆。15个乡镇文化站、58个社区图书室和农家书屋建成。体育事业成绩喜人，积极开展全民健身运动，安装体育健身路径器材200多套。竞技体育水平不断提高，获省级以上赛事金牌355枚，创我市历史最好水平。2010年，国民体质监测站、国家级短跑人才基地建成使用。承办了全国中学生篮球赛，鹤岗一中获得冠军。在全省第十二届运动会上，取得团体总分第五名的好成绩。

　　过去的五年，是人民群众得到实惠最多的五年。为人民群众办实事、做好事的数量逐年增加，并上升为“工程式”推进。其中，投资百万元以上的事项五年达到374项，累计投入资金超过138亿元，是“十五”期间的30倍。在岗从业人员达到27万人，比“十五”期末增长4.5%。累计发放城乡低保资金9.8亿元，是“十五”期间的3.2倍。人均住宅建筑面积从21.54平方米增至24.84平方米。完成了80个村3.2万人整村脱贫任务。2010年，我市“十项工程”共实施154件惠民利民的大事实事，其中，已有116件完成或超额完成，38件跨年度实事顺利推进。市级财政用于民生和社会事业项目资金达到17亿元，是投入最大的一年。积极促进就业创业，我市被省政府确定为首批“创业型试点城市”，“百名大学生就业创业工程”和妇女创业就业贷款贴息政策等全部落实，全市实现新增就业再就业2.26万人，城镇登记失业率控制在4%以内。不断完善社会保障体系，提高各类补助标准，企业职工失业保险标准由260元提高到300元；企业退休人员养老金标准由1040元提高到1164元；城市低保人均补差额由173元提高到203元，列全省第三位；农村低保人均补差额由900元提高到1200元，列全省第二位。将3.5万户低保和低收入家庭纳入医疗救助和保险范畴；将1.75万名“家属工”和“五七工”全部纳入养老保险范畴；争取6300万元资金，将六区7300名国有和集体困难企业退休人员纳入基本医疗保险范畴；将2700名“老工伤”人员全部纳入工伤保险范围；为1万余名企业并轨人员解决了失业保险金问题。为6700名60周岁农村老人发放基础养老金445万元，为4370名城市80周岁以上低保、低收入老人和90周岁以上老人发放生活津贴262万元。城镇居民医疗保险扩面达到19万人，覆盖率达到70％。大力实施安居工程，总计107万平方米涉及1.6万户的沉陷区治理任务全部完成；总计477万平方米涉及7万户的棚户区改造工程，已累计开工117万平方米，竣工48万平方米；总计154万平方米涉及1.66万户的农村泥草房改造工程，已累计完成8987户、61.87万平方米，完成总任务的54%；总计8.3万平方米涉及1700多户的国有林场危旧房改造工程开工建设9600平方米；建设或购买廉租房4048套16.8万平方米，已分配585套；租房补贴范围达到1.46万户，累计发放住房补贴4409万元。首期一氧化碳渗出区居民整体搬迁完成年度任务。积极创建平安城市，连续三年开展“安全生产年”活动，治理各类安全隐患9800多起。城市消防连续6年保持无人员伤亡纪录，15年无重特大火灾事故，被公安部评为全国示范单位。在2009年全市综合安全生产工作和地方煤矿安全生产双获第一的基础上，2010年再度被省政府评为“安全生产优秀单位”。高度重视社会稳定，近百件信访积案得到彻底解决。妥善处置“集资借贷”事件，累计还款（含实物折算）近22亿元，约占集资总额的59%；累计对1万多人次发放大病治疗和子女就学救助金5500多万元，得到国家有关部门和省委、省政府充分肯定。切实加强社会治安综合治理，先后开展了打黑除恶、打击黑车等30余次专项活动，打掉了两个37人“涉黑”团伙。建成了第二看守所、第四消防队，购置了公安应急通讯指挥车、大吨位高压消防车等装备。开通了城市监控网络体系，城区新装治安监控摄像700多个，重点部位覆盖率达到80%。治安形势稳定好转，刑事案件发案率下降8.4%，破案率上升12.3%，现行命案破案率首度实现100%，我市实施的侦破命案“五大战法”在全国推广。此外，我市近年来还以民生为重点，投入4350万元设立了21项保障性基金，其中，2010年投入1200万元，新设了公共卫生应急、政法干警救助、信访维稳救助和涉诉群众司法救助等7项基金。

　　过去的五年，是改革开放逐步深化的五年。积极推进政府管理改革创新，完成了政府机构改革，启动了政府效能建设。制定出台了《鹤岗市城市资源经营管理办法》、《鹤岗市人民建议征集和奖励办法》、《鹤岗市市民特殊荣誉奖励办法》、《鹤岗市政府工作创新奖评选办法》等35个制度性文件。努力提高决策水平，组建了市政府项目建设顾问组和城市规划专家组；不断提高行政审批效率，建立了人民办事中心，减少了86项审批事项。财政、金融等方面改革稳步推进，金融支持地方发展力度明显增强，银行存贷款余额分别比“十五”期末增长1.2倍和1倍。对外开放实现新突破，与俄犹太州及比罗比詹市友好关系不断巩固，对俄合作项目累计达到41个。2010年，赴俄森林采伐完成1万立方米，土地种植达到8300公顷，生猪养殖存栏3300多头。年产40万吨锰矿开发项目即将开工剪彩，冬季过境固冰浮桥项目经两国交通分委会批准并开始规划设计。与27个国家和地区开展了贸易往来，4户企业取得了外贸经营权，并对爱尔兰、芬兰、挪威等国实现了“零”的突破。进出口总额完成8180万美元、利用外资完成3320万美元，增幅列全省第四位，分别是“十五”期末的5.7倍和19.7倍。国内合作不断拓展，与省内外50多个商会建立了联系，与北京海淀区、朝阳区友好关系不断加强，同湖北恩施和仙桃、吉林通化，以及东北东部十二市（州）等地签订了合作协议，等等。

　　此外，民族、宗教、妇儿、外事、侨务、审计、监察、物价、人防、司法、统计、地震、残联、老龄、档案、无线电等项事业都取得了新成绩。

　　各位代表，回顾“十一五”的发展历程，我们欣慰地看到，这五年是我市改革开放以来发展最好最快的五年，也是为长远发展奠定重要基础的五年。特别是2010年成绩的取得，是在鹤矿公司限产减产原煤240多万吨，新兴矿尚未恢复生产；华能鹤电公司受煤价、发电等因素制约，亏损近亿元；市内近80家企业受“集资借贷”严重冲击，不能正常生产经营并享受扶持政策；我市多年没有铁路、公路、机场等重大基础设施项目投资，大多数产业项目尚未形成财力等诸多不利因素影响的情况下完成的。因此，这些成绩来之不易、弥足珍贵。这些成绩的取得，是省委、省政府和市委正确领导的结果，是人大、政协监督支持的结果，更是全市人民凝心聚力、团结奋斗的结果。在此，我代表市政府，向全市各族人民、各民主党派、工商联、各人民团体和各界人士，向中省直各单位、驻军官兵和所有支持帮助鹤岗发展的各级领导、各方友人致以最诚挚的感谢和最崇高的敬意！

　　回顾“十一五”的发展历程，我们还必须清醒地认识到，我市在经济社会发展中还存在许多矛盾和问题：一是在经济发展方面，经济总量较小，且增长过度依赖投资和资源，产业结构层次较低，发展方式相对落后，人才科技支撑能力严重不足。二是在财源建设方面，新上税源项目大多处于建设期和政策优惠期，短期内还不能对税收、就业等形成有效拉动，财政压力增大。三是在城市建设方面，基础设施欠账较多，城市管理水平亟待提高，特别是供热、供水、供气能力和背街小巷、居民区内环境治理亟待加强。四是在改善民生方面，城乡人民收入水平总体偏低，贫富差距较大，财力与需求的矛盾更加突出。五是在安全稳定方面，基础、基层工作薄弱，投入不足、标准不高、隐患整改不到位等问题仍然存在，特别是煤矿安全、“集资借贷”问题仍然压力重大。六是在政府自身建设方面，职能转变较慢，发展环境仍需改善，一些职能部门办事效率低、服务质量差，个别公职人员弄权勒卡损害群众利益的问题仍然存在，让群众不满甚至痛恨的事情仍时有发生。对这些问题，我们必须高度重视，抓紧解决。

　　二、“十二五”时期和2011年主要任务

　　“十二五”时期，既是我市释放潜力、加快发展的机遇期，也是改革开放、跨跃赶超的攻坚期。面对各地激烈的竞争和相对落后的现实，全市上下务必保持清醒的头脑和坚定的信心，坚持团结一致、艰苦奋斗，努力使这五年成为我市经济社会发展、城乡面貌变化和人民生活改善更好更快的时期。全市经济社会发展的主要目标是：确保实现“三个翻番”，即地区生产总值和财政收入分别比2010年翻一番，固定资产投资累计比“十一五”时期翻一番。在此基础上，力争实现“712”目标，即地区生产总值达到700亿元、财政收入达到100亿元、固定资产投资累计达到2000亿元；同时实现城镇居民人均可支配收入和农民人均纯收入翻一番。

　　2011年是实施“十二五”规划的开局之年，做好今年的各项工作，对今后五年具有重大意义。今年政府工作的总体要求是：全面贯彻落实中央十七届五中全会精神和省委“八大经济区、十大工程”的战略部署，坚持以科学发展为主题，以加快转变经济发展方式为主线，坚持“五先一创建一全力”的发展战略，突出项目产业建设，加快经济结构调整，着力推进改革创新，确保城市稳定平安，努力实现跨跃发展，为顺利完成“十二五”规划，全面建成小康社会奠定坚实的基础。

　　今年全市经济社会发展的主要目标是：地区生产总值增长13%，地方财政收入（按可比口径）增长17%，固定资产投资增长35%，工业增加值增长22%，社会消费品零售总额增长16%，进出口总额和利用外资分别增长15%，城镇居民人均可支配收入增长10%，农民人均纯收入增长15%。同时，财政收入占地区生产总值的比重、税收占财政收入的比重、产业项目投资占固定资产的比重、居民收入占收入分配的比重等体现发展质量和效益的指标要稳步提升。

　　为实现上述目标，要重点抓好六个方面工作。

　　（一）更加注重项目建设，突出优势产业加快发展。项目是最现实的经济增长点，产业是最有效的城市竞争力。从今年开始，要连续五年开展“项目产业建设年”活动，继续开展“四比竞赛”活动，全力推进“五大战略产业、十大重点产业”发展，努力实现由单纯抓项目向重点抓产业转移，由一般性抓产业向抓产业链转移，切实加大产业结构调整力度，着力构建优质高效的现代产业体系。今年，要以大批量建成投产为目标，重点实施100个千万元以上产业项目。煤电化工产业，要以加快现有项目建设速度和延长产业链为主攻方向，重点加快龙煤东化、中海油华鹤两个化肥项目建设进度；宝源、征楠焦化项目完成主体工程，万昌焦化及吉林天富、上海汉兴、北京奥德等焦化下游产品开发项目开工。鹤矿集团城市60万千瓦热电联产、立达矸石发电二期争取开工，鸟山煤矿加快建设，400万吨洗煤项目投产。优质绿色食品产业，要以“吃干榨净出精品”为主攻方向，重点加快兴汇玉米二期、万源粮油、人和米业、泰丰大米蛋白、三江平原米业、绥滨酒业、宝泉酱业扩产改造，以及阿凌达、家富、信赢养殖加工和台湾农民创业园等项目建设，力争年内全部投产。石墨产业，要以实现精深加工为主攻方向，力争香港南海油、山东新华锦、溢祥石墨等项目年内投产，深圳宝安集团石墨深加工项目开工，中铁资源集团等石墨项目完成开工准备。机械加工制造产业，要以打造现代机械装备制造产业体系为主攻方向，重点加快台湾华升等矿用救生舱、世达联合收割机、龙跃玉米剥皮机、鹤峰粮食机械加工、丰屹和弘吉矿山配件等项目建设，力争年内投产。林木加工产业，要以引进大企业、实现深加工为主攻方向，重点推进翔鹤实木家具、富鑫实木门、大兴密度板、广亿工艺家具等企业扩产升级。完成4000亩林浆纸原料基地、5000亩野生蓝莓基地、5000亩红松坚果林基地、1000万袋食用菌基地建设。同时，加快万寿菊、五味子、山野菜、山野果等产品开发。医药产业，要以提升企业效益、壮大产业规模为主攻方向，重点要加快现有优势产品品牌的市场拓展和中药种植加工基地建设，扩大医药特色园区，培育三精、双兰星等骨干企业，加快推进宝泉七味刺榆颗粒、圣峰金胆康胶囊、青龙衣抗癌粉针剂、龙宝抗艾滋病毒药物等项目建设，积极发展生物医药产业。节能环保产业，要以加快循环经济、低碳经济发展为主攻方向，重点推进农研院玉米秸秆循环利用、经纬木糖二期，以及人和、泰丰、三江平原稻壳发电等项目建设，天坤和环瑞煤矸石砖项目投产。同时，加快推进龙源解放山、鹤北杜家山、萝北沙田山风电和宝泉岭国能秸秆发电等项目建设。现代服务产业，要以提升产业比重、改善消费环境为主攻方向，重点打造“国际界江生态游”品牌，着力提升“食住行游娱购”综合配套体系。启动建设旅游集散中心，加快推进名山旅游名镇等景区建设，完成岭北矿山地质公园、太平沟黄金古镇、延兴岛野生动物保护区、边防军事旅游开放区等项目建设。筹备举办第二届国际界江旅游节。同时，举办首届“美食节”。争取大润发超市、红星·美凯龙二期和顺达、南翔、检顺三个物流园区竣工，大商新玛特项目启动。对外合作项目，要以扩大对俄经贸合作、促进进出口增长为主攻方向，完成《对俄边境合作加工区规划》，完善名山口岸设施。积极推进赴俄矿产资源开发、过境种植养殖、房地产开发，恒新节能灯具项目正式启动；鹤矿锰铁矿开发项目开工，境内铸成铁合金项目完成审批；对俄贸易中心、驻俄商务处新楼竣工营业；争取固冰浮桥项目早日开工。与此同时，要积极谋划生成一批符合国家支持政策的新兴产业项目。为保证项目产业建设年开局见效，特别是完成与省政府签约的重点项目建设任务，要进一步强化领导干部包保责任制。全市各单位要切实加大招商引资力度，进一步提高项目建设工作在年度目标考核中的比重。

　　（二）更加注重园区建设，突出提升产业项目承载能力。坚持园区牵动战略，加快全市主体功能区规划建设，努力实现人口、资源、要素、产业、环境科学布局。在全面抓好“省级工业示范基地”建设的基础上，重点加快“两城一带十大园区”建设。“鹤南工业新城”，要以兴安区为主体，整区定位为工业区，版图由现有的16平方公里扩大到100平方公里左右，今年完成整体规划和行政区划调整，开工建设重点工程项目。“中国石墨城”，要以萝北石墨园区为主体，充分发挥优质石墨资源储量亚洲第一的优势，引进大企业、大集团和研发机构，力争早日建成全国最大最优的石墨产业基地。“东宝鹤萝综合产业带”，要以东山区、宝局、鹤北局、萝北县为主体，利用国家支持我市“接替产业集聚区”建设和兴建鹤名公路、鹤名铁路的机遇，充分发挥大企业集合优势，积极推进产业项目和园区集聚发展。“十大园区”，要以现有市企县区的特色产业园区为主体，加快提档升级，力争做大做强做优。同时，积极推进与上海市松江区机械制造产业转移基地、宁波圣罗集团棉纺织品生产基地、中海油北方煤化工产业基地建设。要加快园区供水、供电、运输等基础设施建设，开工建设工业示范基地综合服务中心，进一步提高园区配套能力和项目竞争能力。

　　（三）更加注重基础设施建设，突出加快现代农业发展。按照中央和省委农村工作会议部署，今年重点实施“一兴两化、一调两增”战略，积极推进“三江平原农业综合开发实验区”建设。“一兴”即大兴水利。抓住国家大规模投资水利基础设施建设的机遇，完成全市水利工程建设规划，组建水务投资集团和供排水集团。重点完成江萝灌区干渠、德龙灌区渠首、小鹤立河水库扩建工程和阿凌达湖除险加固等工程。抓紧完成金顶山中型水库、关门嘴子水库前期工作，争取早日开工；跟踪推进太平沟水利枢纽工程。新建4座塘坝，完成灌区田间配套工程和涝区清淤整治工程，增加旱涝保收田6万亩。“两化”即农业机械化和产业化。实施“农业机械化整市全程推进工程”，争取农机补助资金5000万元，新组建5个现代农机合作社。实施“农业产业化升级工程”，新增龙头企业5户，争取做到玉米、大豆原粮不出市，水稻外购量超过本地产量，企业销售收入和利税分别增长10%。“一调”即调整优化种植结构。重点实施“水稻过半工程”，今年新增水稻面积30万亩，争取两年达到耕地总面积的50%。有机、绿色、无公害食品种植面积达到160万亩；杂粮、果蔬、食用菌、饲草等优质高效作物达到8万亩。“两增”即农业增产、农民增收。进一步调动农民种粮积极性，加速推进农业科技化、标准化和服务社会化，提高粮食产量和效益。切实抓好畜牧水产养殖基地建设，牧（渔）业产值和增加值分别增长8%、7%。同时，要积极引导农民发展劳务经济和多种经营，确保实现农民增收目标。

　　（四）更加注重协调发展，突出增强区域整体经济实力。进一步加快区域经济发展。充分发挥域内鹤矿集团、宝泉岭农垦分局、鹤北林业局、华能鹤电公司等大企业的优势，建立合作机制，落实优惠政策，努力实现市企在城市建设、项目建设、园区建设、旅游开发等方面共建共享、互利互惠，联手提升区域综合竞争力。进一步加快县域和城区经济发展。增强县区统筹发展能力，加速实现工业化、产业化、城镇化和城乡一体化。坚持责权利统一原则，制定实施区级职能职责调整方案，着力解决制约县区经济发展的体制机制问题，发挥好、保护好县区发展的积极性和创造性，不断提高县区整体竞争力。进一步加快非公有制经济发展。认真落实《关于促进非公有制经济加快发展的若干意见》，对非公有制经济实行“非禁即入”原则，着力解决制约企业发展的资金、运输、环境等实际问题，提高企业运行质量和效益，不断提高企业核心竞争力。争取非公经济规模以上企业增加到150户以上，增加值增长20%。

　　（五）更加注重改善民生，突出抓好十大工程。坚持把改善民生做为政府工作的根本目标，把人民群众满意做为政府工作的根本标准。无论克服多大困难，都要确保鹤岗的各项民生工作不落在全省13个市地的后面。今年，要以人民群众最关心、最直接、最现实、最急需的问题为重点，进一步加大投入力度，切实抓好137件大事实事。一是抓好文明城市建设工程。以年底基本达到“三优”文明城市标准为目标，以“城市管理年”活动为载体，继续加快城市建设步伐，加大物业、交通、市场、用地、公共资源等经营管理力度，着力解决城市供水、供热、清雪、卫生、动迁、回迁特别是“脏乱差”等群众反映突出的问题。加快交通网络建设，重点是完成全市交通规划布局，鹤名公路全线开工，哈佳鹤城际铁路、鹤名铁路加快推进。完成富绥松花江大桥引道和路面工程，修建5条城市道路，加快背街巷道升级改造，基本完成通村公路建设任务，启动绕城公路、城市高架桥、鹤伊高速公路、绥萝公路升级、新火车站和机场等项目前期工作。坚持优先发展公共交通，更新20辆公交车，实现公交线路向工业园区、新建小区和旅游区延伸。加强各种运营车辆管理，及时解决出租车的实际问题，严打各类“黑车”和违法经营。加快城市“五化”建设，硬化要重点向居民区延伸，在居民庭院新建或改造100个小广场、小绿地、小花园，全部完成10万平方米裸土治理任务。绿化要坚持能绿尽绿、因地施绿，科学种植乔灌花草，积极开展种植“公仆林”活动，完成150万株义务植树任务。亮化要进一步提高标准，推广节能，完成城市新建道路、重点桥梁和主街两侧楼宇亮化。美化要全面提升各类建筑物设计水平，完成振兴广场扩建和“五山”基础设施改造，完成城市主要街路两侧建筑和8个住宅小区200万平方米既有建筑节能美容任务。净化要重点强化城市日常管理机制，抓好垃圾、冰雪清运工作，提高环卫机械化水平，引进市场机制。加快“四供两治”项目建设，提升城市供热能力，确保供热达标，对“弃供”企业实行“终身禁入”；争取开工建设城市北部供热项目；实施可再生能源供热项目，新增2000万平方米房屋建筑供热能力。增加城市气源工程，加快龙煤集团、大庆油田公司煤层气等项目推进工作。提高饮用水质量和项目用水保障能力，完成供水管网改造二期工程，启动饮水困难地区供水改造，推进农村饮用水安全工程。加快鹤岗、兴山、峻丰、绥滨输变电工程和新一轮农村电网升级改造工程建设。加快“滨水城市”建设，完成清源湖、天水湖、鹤立湖、阿凌达湖、松鹤西湖景区基础设施主体工程，启动小鹤立河流域治理工程，开工建设“鹤南滨水景观带”，建成石头河、西山沟等防洪工程。加快小城镇建设，完成全市重点小城镇建设规划，忠仁镇、新华镇、五道岗农场等重点工程开工。同时，要不断提升城市文明程度和市民文明素质，制定实施“三年文明建设规划”，开展“热爱鹤岗、建设鹤岗、发展鹤岗”系列活动。二是抓好教育优先发展工程。以促进教育公平、提高教育质量为核心，科学配置教育资源，努力提高各级各类教育综合发展水平。积极发展学前教育，新增6所、改善2所公办幼儿园，支持开办私立幼儿园，并达到标准化水平。创建10所省级标准化学校和5所标准化样板学校。建成市职教中心教学楼、卫校实验楼、一中音乐厅、八中综合楼和2所中小学校外活动中心。推进优质特色高中建设，启动国家级重点中等职业学校创建工作。推进校舍安全工程，加快学校供热网线等基础设施改造。逐步提高学校班主任补贴待遇，从每月14元提高到100元。全面贯彻落实经济困难学生资助政策，提高农村义务教育困难家庭经济困难寄宿生生活费补助标准，小学、中学每生每年由500元、750元，分别提高到750元、1000元；普通高中家庭经济困难学生资助标准由每生每年1000元提高到1500元，资助比例由15%提高到20%左右；将中等职业学校城市家庭经济困难学生纳入免学费范围，确保全市经济困难家庭子女全部正常上学。三是抓好医疗卫生服务工程。以加快医疗卫生体制改革，提高医疗水平为核心，以解决“看病难、看病贵”为重点，全面提升公共卫生服务能力和突发事件应急处置能力。完成市医疗中心综合大楼主体工程，建成职业病防治院和中医院中药制剂室，启动妇幼保健院综合楼迁建等工程。加快推进县、乡、村和社区医疗卫生机构建设，进一步加强在职医务人员的培训。全面落实“婴幼儿健康促进计划”、青少年近视眼防治、结核病体检筛查和农村35至59岁妇女“两癌”检查等免费救助政策，继续实施人口与计划生育“惠家工程”。为65岁以上老年人实施免费体检并开设专用服务窗口。城乡居民医疗保险财政补助水平由120元提高到200元，人均基本公共卫生服务经费标准由15元提高到25元。全面实行公立医院预约诊疗和网络远程会诊服务制度，建设药品集中招标采购配送中心。推行市级定点医疗服务机构垫付结算制度，实现“一卡就医、即时结算”。落实医疗卫生机构基本药物制度，实现药物价格合理，保证用药安全。四是抓好文化体育繁荣工程。加强公共文化服务体系建设与改革，丰富城乡群众精神文化生活，推进文化、体育产业化发展。完成市博物馆主体工程和电视台新闻演播厅改造工程。新增数字电视用户4万户，数字化率达到50%。建成市文化活动中心、老年大学及数字电影院、文艺作品展厅等，实现全市所有乡镇建成综合文化站的目标。举办“十一五成就和十二五展望”等主题宣教活动，开展“欢乐鹤岗行”文化大篷车活动和“一村一月一场电影”活动。新建30个农家书屋。继续实施《全民健身条例》，建成全民健身主题公园，新增全民健身路径器材30套、农民健身工程10项。举办市第九届运动会。五是抓好人才科技强市工程。全面加快人力资源开发步伐，大力培养引进产业建设、金融管理、城市规划、旅游开发等方面实用人才，着力解决人才严重匮乏问题。充分发挥科技先导作用，不断提升自主创新能力。开展优秀拔尖人才评选活动；建成“黑龙江省东部煤电化高级人才培训基地暨黑科大研究生分院”，首批硕士研究生招生开班；建成市青少年科技活动中心和“三检大厦”，开工建设食品药品、保健品和化妆品检验检测中心。支持企业建立科技研发机构和产业技术创新战略联盟，新增国家高新技术企业2户，创建民营科技企业6户。建成50个农业科技园。与省科技厅建立科技合作共商机制，大力转化和引进科技成果。六是抓好生态环境保护工程。切实加强森林、湿地、江河、山体、地质遗迹和动植物保护等生态体系建设，进一步优化生态环境。严格执行项目节能环评制度，建立生态补偿机制，为可持续发展预留环境空间。财政安排1000万元专项资金，支持节能减排工程项目建设。按期完成松花江流域治理项目和节能改造任务，实现华能鹤电脱硫工程和西部污水处理厂稳定达标运行。建成城市东部污水处理厂、垃圾处理厂和医疗污水处理厂，启动萝北、绥滨两县污水、垃圾处理工程和向阳区垃圾填埋场环境治理工程。关闭各类严重污染企业，彻底拆除原鑫塔水泥厂和市郊白灰厂。实现二氧化硫排放量继续下降，化学需氧量降低2%，单位生产总值能耗下降4.5%的目标。七是抓好人民群众安居工程。以全面改善人居环境，解决中低收入群体住房为重点，加快“两棚三房”改造力度，力争用三年左右时间基本解决全市居民住房困难问题，努力实现“住有所居、居有改善”。完成煤矿棚户区改造82万平方米住宅小区和配套公建工程，安置1.4万户居民。启动城市旧区棚户区改造，对易灾区和大多数群众支持改造的区片实施优先改造。集中建设廉租房1000套，并安排100户计划生育特困家庭入住廉租房；为1.46万户居民发放租赁住房补贴。启动建设公共租赁房项目。完成农村泥草房改造3900户，农村砖瓦率达到84%，力争全部完成农村重点优抚对象泥草房改造。完成1.2万平方米林区危旧房改造工程。完成首期一氧化碳渗出区居民整体搬迁，启动南山、东山地区渗出区治理工作。在各类居民住房建设中，要坚决遏制低水平建设，所有项目必须保证质量安全和基本服务功能，并注重外观和环境美化，严防出现“楼房棚户区”造成巨大浪费。八是抓好就业创业增收工程。把就业创业摆上更加突出的位置，抓好“省级创业就业试点市”建设，不断扩大公共就业服务力度。着力提高低收入阶层收入水平，关注解决中下等阶层的实际困难，不断扩大“中产阶层”，普遍提高城乡居民收入水平。全年实现新增就业2.3万人以上，安置再就业2000人以上，城镇登记失业率控制在4.3%以内，保持“零就业家庭”动态为零。加强特殊群体就业救助，实施高校毕业生就业创业支持政策，力争使85%以上的高校毕业生实现就业；为有创业愿望人员发放小额担保贷款1000万元。完成农村劳动力转移6万人次，实现劳务收入2.5亿元。大力开展职业技能和创业培训，全年培训5000人次。实施创业带就业三年行动计划，积极发展家庭服务业，培植一批小老板、小业主，对新办小企业实行税费减免政策。实施“促进青年就业创业十百千援助工程”和妇女创业小额贴息贷款政策，建成鹤岗家政网络服务中心。建立健全企业工资正常增长机制，最低工资标准提高10%以上。提高社区工作者生活待遇补贴，正主任从600元提高到1000元，副主任从500元提高到900元，退休社区主任每人每月平均提高100元，达到全省中上等水平。全额兑现机关事业单位人员津贴补贴。城镇居民可支配收入两年超过全省平均水平，农民人均纯收入两年达到1万元。认真解决拖欠农民工等职工工资问题，力争实现集体访和越级访为“零”。九是抓好社会保障扩升工程。继续实施“五险”扩面，不断完善救助体系，努力实现“应保尽保”。城乡居民最低生活保障标准，每月人均分别提高20元和15元，保持全省前三位水平；企业退休人员养老金标准由1090元提高到1240元；失业保险金待遇从300元提高到490元;争取1亿元资金，将2万名关停破产企业的退休人员纳入医疗保险范畴。积极解决农林牧渔四场、刑满释放劳教人员等参加养老保险问题、城镇老年居民基本养老保障问题、企业老工伤人员待遇问题，以及被征地农民社会保险与城镇养老保险政策衔接等问题。农村五保供养补助标准超过全省平均水平。加快发展养老服务事业，抓好养老机构建设，每个乡镇至少建立1个居家养老服务示范点，养老床位达到4000张。深入开展“慈善超市进社区”活动，完善30个慈善超市建设。进一步提高儿童福利机构供养及社会散居孤儿生活补助标准，建成残疾人综合服务中心。全面实施“金保工程”，实现新增参险人员1万人以上。改善现有殡仪馆服务条件，建成兴安殡仪服务站。全面加强和谐社区建设，实施专业化社工服务“百千万”工程，改扩建30个社区综合服务站，创建3个民政服务示范县区、10个标准化示范社区和3个农村样板社区。完成16个村整村扶贫开发，实现9340人脱贫。投入1000万元，对困难群众开展“情暖万家”大型救助活动。切实加强物价监管，投入600万元调节基金，对低收入群体进行物价补贴。切实落实“米袋子”、“菜篮子”行政首长负责制，制定《鹤岗市蔬菜生产实施方案》，抓好蔬菜生产和仓储设施建设，建成500栋蔬菜温室大棚，蔬菜种植面积达到2万亩，产量达到8万吨，努力让群众吃上低价菜和放心菜。十是抓好平安城市创建工程。全面加强安全生产工作，深入开展“安全生产年”活动。坚持以煤矿安全生产为首要任务，严格落实政府和企业两个主体责任，加快推进机械化、标准化、数字化建设和集团化经营。全面开展食品安全、校园安全等专项整治工作，坚决遏制重大事故发生。坚持把依法处理“非法集资”事件作为社会稳定的首要任务，进一步加快还款进度。对严重违法的企业法人坚决依法处置；对积极还款的企业继续实行扶持政策；对困难人员继续实施救助政策，力争到年末我市“集资借贷”处置工作完成阶段性任务。建成“人民诉求来访调处中心”，实行“一站式”服务、重点部门集中接访、领导干部定期接访、基层组织调处等制度，妥善解决群众诉求问题，努力促进社会和谐。建成城市应急指挥中心、市气象观测站和区域雨情、水情观测站；完成煤矿安全避险六大系统建设和煤矿产量监控系统建设，加强国家级救援基地建设。继续保持对各种违法犯罪的严打高压态势，增加公安、武警、消警等各警种设备设施投入。建成互联网管控系统，购置网络移动定位车、光检车等设备。完成市县两级应急通信系统、交巡警指挥中心、交通安全教育基地等项目建设；建成第一、第二看守所和拘留所“三所”合一的监管专区。加快推进“法眼工程”，新装400个监视器，城区重点部位覆盖率达到90%。开展安全文化“四进”活动，不断增强人民群众的安全感。

　　（六）更加注重政府自身建设，突出提高领导科学发展能力。新的形势和任务，对政府工作提出了更高的要求。我们必须致力发展、强力争先，全面开展“学先进、找差距、搞对标、促发展”活动，努力实现各项工作在全省晋位升级。对连续两年排在全省末位的单位主要负责人进行行政问责；必须尊重群众、服务群众，秉持公平正义，高度重视民情民意民声民忧，把能为老百姓办实事、解难题，作为衡量公职人员特别是领导干部能力、作风优劣的重要标准。对连续两年群众满意度排在末位的单位主要负责人进行调整；必须勤政廉政、转变作风，坚持重实干、重落实、重实效，坚持“一岗双责”，恪守廉政准则，对各类腐败问题和损害群众利益、破坏发展环境的公职人员进行严肃查处；必须加强学习、强化本领，切实提高执行力，把握主动权，增强创造性，深入开展全面工作“提质提速年”活动和重点工作“倒计时推进落实责任制”；必须转变职能、接受监督，建成公共资源公开交易中心和电子监察网络平台，促进行政权力规范、透明、高效运行，自觉接受人大、政协和广大人民群众的监督，不断提高政府公信力；必须科学理财、勤俭持家，进一步加强财政金融工作，着力建设“诚信鹤岗”，切实加大金融支持地方经济社会发展的力度。严格控制会议费、差旅费、接待费、公车费及各种表彰评比活动，力争实现各类公用经费“零”增长。总之，一定要努力把本届政府建设成团结、务实、干净、高效、为民的政府。

　　各位代表！蓝图已经绘就，目标已经明确，鹤岗已站在新的历史起点，进入新的发展阶段。我们决心在省委、省政府和市委的正确领导下，在全市人民的大力支持下，不惜心血，不辱使命，奋发图强，励精图治，为全市人民生活得更加富裕、更有尊严，作出无愧于历史的贡献！
